# The hidden power of antisense long non-coding RNAs: a dive into a novel regulatory layer mediated by double-stranded RNA formation

**DOI:** 10.1080/15476286.2025.2530797

**Published:** 2025-07-09

**Authors:** Jan-Philipp Lamping, Heike Krebber

**Affiliations:** Abteilung für Molekulare Genetik, Institut für Mikrobiologie und Genetik, Göttinger Zentrum für Molekulare Biowissenschaften (GZMB), Georg-August Universität Göttingen, Göttingen, Germany

**Keywords:** asRNAs, double-stranded RNAs, dsRNAs, SUTs, lncRNA, gene expression, RNA export, adaptation, translation, pervasive transcription

## Abstract

Over the past decade, non-coding RNAs (ncRNAs) have gained prominence in research due to their widespread presence in cells, yet their functions remain increasingly complex and less understood. Despite being initially deemed ‘junk’, many lncRNAs are now recognized as key regulators in cells and are often affected in disease contexts. Notably, numerous mRNAs have annotated antisense RNAs (asRNAs). Because asRNAs resemble the largest group of lncRNAs and were identified to serve a general function in *Saccharomyces cerevisiae*, they are the focus of this review. In *S. cerevisiae*, the absence of RNA interference (RNAi) enables unbiased study and allowed researchers to investigate their roles in gene regulation more directly with intriguing results, summarized here. Expression of asRNA leads to the formation of double-stranded RNAs (dsRNAs) with the regarding sense counterpart, resulting in enhanced gene expression through preferential nuclear export. Thus, these hidden leaders can boost gene expression and require future attention pivotal for elucidating their influence on biological processes and revealing disease mechanisms.

Over the past decade, non-coding RNAs (ncRNAs) have gathered significant research interest due to their abundant presence in all types of cells. However, despite extensive research, the functions of ncRNAs have only gradually started to become better understood. This review summarizes current knowledge of long (l)ncRNA with focus on antisense RNA (asRNA) functions in eukaryotes. We focus on *Saccharomyces cerevisiae*, as baker’s yeast has lost its RNA interference (RNAi) system in evolution and therefore lncRNAs were investigated unbiased.

Generally, ncRNAs are classified into two groups based on their length: the small (s)ncRNAs ranging below 200 nt and the lncRNAs which exceed 200 nt. sncRNAs include the well-described snoRNAs, snRNAs and tRNA. In contrast, lncRNAs are more diverse and not easy to classify into functional groups. Initially, lncRNAs were rather defined as junk transcripts due to their overall short half-life in cells [1]. However, since then, multiple functions in *cis* and *trans* have been described across many organisms for single genes, including transcriptional repression, chromatin remodelling, regulation of histone modifications, post-transcriptional regulation, RNA processing, RNA stability and modulation of translation [2–11]. Importantly, dysregulation of multiple lncRNAs was shown to be connected to diseases such as cancer and neurodegenerative diseases and many more [7,8,12]. The presence of over 100,000 annotated lncRNAs, outnumbering protein coding genes, in human cells – along with the continual discovery of new ones – clearly underscores their significance and regulatory potential. This overwhelming diversity paired with their exclusive presence under specific conditions such as in developmental stages, cellular stress situations or in particular tissues, similar to what is observed for stress response specific mRNAs, hints at their executive roles in various biological processes and cellular functions [7,13–15]. Interestingly, while some mRNAs are condition-specific, the ENCODE project demonstrated that lncRNA expression is more cell-type specific, with 29% expressed exclusively in only one cell type and only 10% in all cell lines, whereas 53% of mRNAs show constitutive expression [16]. Their selective presence illustrates their tightly controlled expression as they are not always required [17,18]. Furthermore, it is noteworthy that the number of lncRNAs tends to increase with the complexity of the organism [7,19]. This trend highlights their significance and underscores the necessity for a deeper understanding of their functions and modes of action within various biological contexts. Their number is still vastly growing because it is generally more difficult to predict the existence of a lncRNA ahead of its experimental discovery, because most of them lack a canonical open reading frame (ORF), which is the recognizable feature for an mRNA. Interestingly, the discovery of pervasive translation of many lncRNAs with noncanonical, small (sm) ORFs may be a new handle for their computational prediction [20,21].

## Synthesis and genomic positions of lncRNAs

Eukaryotic genomes exhibit extensive transcriptional activity, designated as pervasive transcription. It refers to the widespread production of RNAs from genomic regions beyond those encoding proteins. Such transcripts comprise 70–90% of the genome. However, the regulation of pervasive transcription remains elusive and needs further research and importantly, not all asRNAs are transcribed through pervasive transcription.

Over the years, the individual roles of several long non-coding RNAs (lncRNAs) have been elucidated, revealing their involvement in various cellular processes. However, it is important to note that only a small fraction of these lncRNAs has been confidently assigned to a specific function, which emphasizes the complexity of lncRNA biology and the need for continued efforts to characterize their diverse roles and modes of action in different biological contexts.

**Figure 1.**
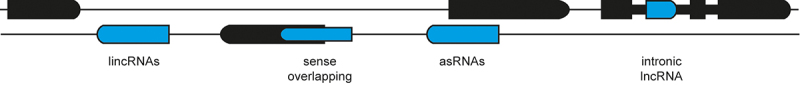
Positions of mRNAs and lncRNAs in the genome. lncRNAs can either be located between genes, designated as lincRNA, or overlapping with the mRNA or arise from intronic sequences. The largest class of lncRNAs are asRNAs.

Generally, lncRNAs are encoded in the genome individually or overlapping with genes in sense or antisense direction (Figure 1). They can arise from sequences located between genes, known as long intergenic ncRNAs (lincRNAs), or can be transcribed in the same direction as a sense gene. Additionally, they can originate from intronic regions or from the opposite strand of a gene, classifying them as antisense RNAs (asRNAs). This latter group represents the largest category of lncRNAs across eukaryotes, with currently approximately 60% human genes possessing an annotated asRNA [9]. In fact, ~40% of the lncRNAs are asRNAs, underlining their potential functional significance in regulating gene expression and contributing to the complexity of genomic regulation in humans and other eukaryotic organisms [15,22,23].

The potential formation of double-stranded RNA (dsRNA) through base-pairing between asRNAs and their corresponding sense RNAs positions these pairs as viable targets for RNA interference (RNAi), leading to their processing into siRNA by the RNase III enzyme Dicer. These siRNAs are subsequently incorporated into the RNA-induced silencing complex (RISC) which utilize them as guides to target and degrade complementary RNA, thereby leading to effective gene silencing. Importantly, not all dsRNAs are prone to direct gene silencing, but might be implicated in other roles and functions.

Given the implications of RNAi in gene regulation, the budding yeast *Saccharomyces cerevisiae* serves as an ideal model organism for studying lncRNAs, particularly asRNAs, because of the absence of a functional RNAi pathway. This allows researchers to analyse the roles and mechanisms of asRNAs without the confounding effects of RNAi-mediated gene silencing. This simplification facilitates a clearer understanding of asRNA biology, their expression patterns and their potential functions in gene regulation and cellular processes.

**Figure 2.**
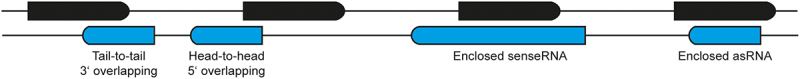
Positioning of asRnas. Sense mRNA and the regarding asRNA can overlap in different ways. It can be distinguished between 3’ and 5’ overlapping sequences, as well as enclosed asRNA or mRNA.

asRNAs can be further characterized based on their spatial relationships to the associated sense transcript (Figure 2). Positional variations of asRNAs, such as head-to-head overlap, tail-to-tail overlap, enclosed antisense and enclosed sense, significantly influence their regulatory mechanisms and functional roles, affecting how they interact with target genes and modulate gene expression [9].

In the head-to-head (HTH) overlap configuration, the 5’ ends of both RNAs overlap, often with a 3’ overhang. This arrangement is typically linked to bidirectional promoters, allowing for the simultaneous transcription of both the sense and antisense strands, but could also arise from internal sites. On the other hand, the tail-to-tail (TTT) overlap arises when the asRNA overlaps with the 3’ end of the sense gene starting from its 3’ end, resulting in a 5’ overhang. This type of overlap is produced from transcriptional activity within nucleosome depleted regions (NDRs) at 3’ untranslated regions (3’UTRs) or from bidirectional promoters. Another interesting configuration is the enclosed antisense, where the asRNA is transcribed from within the coding region of the sense gene, mainly emerging from intronic sequences or 3’UTRs. This positioning can influence gene expression co- and post-transcriptionally [12,24–26]. Understanding these configurations is crucial as they provide insights into how asRNAs may exert their regulatory effects on gene expression. In *S. cerevisiae*, asRNAs that overlap the transcription start site (TSS) of their corresponding sense genes are more likely to play a direct regulatory role in modulating the transcription initiation rate of the regarding sense genes and thereby their expression level similar to what is believed for human 5’ overlapping asRNAs [2,12,23,27–29]. In higher eukaryotes, 3’ overlapping or full overlapping asRNAs are thought to play a role in regulating alternative polyadenylation of its sense partner [12,30]. Although the choice of the polyadenylation site is believed to be rather influenced by epigenetic modifications and chromatin structure than by formation of dsRNA, the different lengths of 3’UTRs impact stability and fate of the resulting mRNA [12,30,31]. Antisense RNAs that are transcribed from a region within the coding region or within introns (enclosed asRNAs) have been implicated in the regulation of alternative pre-mRNA splicing which seems connected to cancer development [12,24–26]. It is unclear how exactly splicing is affected but it is most likely connected to the chromatin state which impacts the recruitment of the splicing machinery or the recognition of splice sites. In that way, asRNAs can alter the final mRNA isoforms produced from the sense gene, leading to diverse functional outcomes [12,32]. Although these associations between asRNA positioning and their regulatory functions are intriguing, it is important to note that conclusive studies are still lacking.

Much of the current understanding on how the transcription of lncRNA influences the expression of sense genes is based on correlational data, and further investigations are necessary to elucidate the specific mechanisms by which asRNAs exert their effects on gene regulation [4,12,15,22,27,33–35]. The dynamics of asRNA expression and their position relative to the mRNA can either result in a positive (concordant) or negative (discordant) regulation of a gene, highlighting the complexity of their modes of action. The regulatory potential of asRNAs has been thoroughly explored; however, until recently, a comprehensive global function for asRNAs was unknown in yeast.

## Classification of lncRNAs in *S. cerevisiae*

Genome-wide microarray and RNA sequencing analyses have demonstrated a significant presence of ncRNAs in *S. cerevisiae* [33,34,36–39]. Generally, in yeast and Mammalia, lncRNAs are present at lower levels than mRNAs, although there are exceptions like MALAT1 which is one of highest expressed RNAPII transcripts in human cells [7]. However, approaches investigating transcription rate rather than transcript levels, as done by native elongating transcript sequencing (NET-seq) or transient transcriptome sequencing (TT-seq), have shown that the actual transcription rate is higher than the corresponding transcript levels in *S. cerevisiae* [15,28,39,40]. These lower transcript levels can be attributed to the instability of lncRNA transcripts. lncRNAs are synthesized by RNAPII, like mRNAs, and mostly initiate from nucleosome depleted regions, which are commonly found at promoter regions or transcription termination sites of genes. Annotations were optimized using CAGE sequencing (Cap Analysis of Gene Expression) to annotate the 5’ ends [41,42]. However, despite these improvements, there is still a need for further fine-tuning of the annotations to enhance their accuracy and reliability.

First attempts to classify lncRNAs in yeast were based on their stability. Some lncRNAs are detectable in wild-type cells and have been defined as stable unannotated transcripts (SUTs) [33]. As being RNAPII transcripts, these lncRNAs typically receive a 7-methylguanosine (m^7^G) cap at their 5’ end and a poly(A) tail at their 3’ end comparable to mRNAs. Therefore, they are usually protected from nucleolytic attacks. Additional studies classified lncRNAs based on their presence under specific conditions, particularly by examining strains with defects in various RNA decay pathways. This approach led to the identification and further classification of several additional categories of lncRNAs. The cryptic unstable transcripts (CUTs) are enriched in cells lacking the 3’ to 5’ exonuclease of the nuclear exosome, Rrp6 [33,43]. Nrd1 sensitive transcripts (NUTs) are present in strains deficient in Nrd1, which also plays a role in transcription termination, nuclear RNA processing and degradation [38,44–46]. Thus, these transcripts are rather unstable as they are targeted for fast nuclear degradation. Additionally, Xrn1 sensitive transcripts (XUTs) increase when the cytoplasmic 5’ to 3’ exonuclease Xrn1 is deleted [37]. Furthermore, meiotic unannotated transcripts (MUTs) that largely overlap with annotated CUTs accumulate during meiosis [47]. Finally, Set2-repressed antisense transcripts (SRATs) are enriched in the absence of the histone methyltransferase Set2, indicating that Set2 is involved in the repression of these transcripts [48]. Both, XUTs and SRATs are degraded in the cytoplasm indicating that they are capable of being exported from the nucleus. Through this classification, researchers have gained a deeper understanding of the diversity and functional roles of lncRNAs in regulating gene expression and RNA stability. However, these classifications should not be regarded as absolute or mutually exclusive categories; they must be approached with caution and certainly require adjustment based on specific experimental conditions. As demonstrated in later studies, some of these transcripts can overlap, describe the same transcript or arise from one another under certain circumstances, which complicates their classification [4,38,41,47–49]. This interconnectedness suggests that transcripts fit into multiple categories simultaneously, emphasizing the complexity of lncRNA regulation and expression. Therefore, a more nuanced understanding of these classifications is necessary, and a more precise nomenclature is needed to accurately reflect the dynamic nature of lncRNA biology in various cellular contexts.

## Nuclear functions of lncRNAs and asRNAs

The presence of CUTs and NUTs can be understood as part of the regulatory mechanisms governing pervasive transcription. As mentioned before, this phenomenon describes the ability of RNAPII to transcribe both DNA strands at opened chromatin as it can initiate transcription from almost any genomic region provided that the chromatin structure is favourable. Remarkably, although CUTs and NUTs are produced by pervasive transcription, they are immediately eliminated within the nucleus, because they are terminated by the exosome connected Nrd1-Nab3-Sen1 (NNS) complex in early phases of transcription, when the C-terminal domain (CTD) of RNAPII is phosphorylated at Serine 5 [44,50]. NNS-termination supports the recruitment of the TRAMP-complex, which acts as co-factor for the nuclear exosome. This was demonstrated mainly for bidirectional promotors to govern directionality of transcription [4,38,44,51–53]. The reason for their vast production and instant elimination remains still mysterious and needs further investigation. Contrary to SUTs, CUTs usually do not receive a protecting poly(A) tail, but a short oligo(A) tail, which makes them accessible for the exosome and favours instant degradation [38,51,52]. The on average longer SUTs and XUTs with around 900 nucleotides (CUTs are on average below 470 nucleotides) [54] are not targeted for nuclear degradation and can reach the cytoplasm. XUTs mainly consist of asRNAs and have been extensively studied to understand their mode of action. Interestingly, loss of Nrd1 or Rrp6 results in extended versions CUTs making these transcripts more likely to have regulative potential if being antisense to an mRNA [4,38]. Since binding of the NNS factors is altered upon changing conditions, this might as well change the expression of CUTs and NUTs, supporting a condition specific role of these lncRNAs, in which these RNAs are stabilized through extended transcript lengths [55–57]. asRNA can function at different cell stages and thus it is essential to distinguish between the effects resulting from the synthesis of the asRNA and the post-transcriptional effects of the asRNA on the mRNA. Early studies primarily observed an anticorrelation between asRNA and sense RNA, suggesting that asRNA reduces the transcriptional activity of the corresponding sense gene, thereby regulating its expression at the transcriptional level [34,58–61]. Therefore, the focus was rather on the effect of asRNA transcription itself causing transcriptional interference. It was suggested that antisense transcription can act as an on/off switch for low expressed genes having rather little effect on highly expressed genes [4,27,34,37,60]. A requirement for a regulative function of the asRNA on transcription appears to be an overlap with the TSS of the sense gene [4,27,37,60,62]. This switch like behaviour has also been observed in RNA localization studies where sense/asRNAs were expressed in different cells and not simultaneously [4]. Interestingly, the upregulation of either the sense or asRNA was rather suggested to be associated with an increase in the number of cells expressing the respective RNA than an increase in the expression levels within individual cells [4,63]. However, single cell sequencing revealed that co-expression of sense and asRNA is present but accounts only for 10% of the cells in a population [61,64]. The unique presence of an asRNA in a cell indicates that each cell has distinct needs, which draws the attention of regulators towards asRNA. In line with this, antisense RNA transcription overlapping the TSS of the corresponding sense gene has been shown to facilitate the recruitment of components from the histone deacetylase (HDAC) complex and/or the histone methylase Set1 [4,65]. This recruitment alters the histone structure and turnover rates, leading to more closed promoters characterized by smaller nucleosome-free regions. Consequently, this has been suggested to contribute to the repression of sense transcription [2–4,27,59,65].

**Figure 3.**
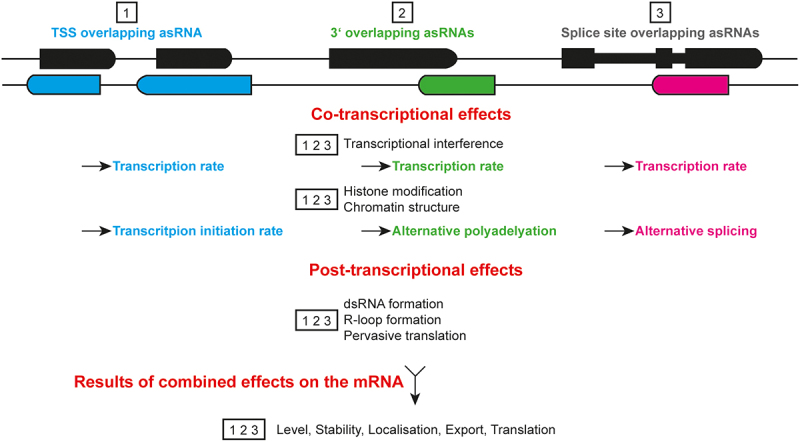
Potential impact of the asRNAs on their complementary mRNAs. Position of the asRNA relative to its complementary mRNA is shown. Potential co-transcriptional and post-transcriptional effects are indicated for each group as well as the potential resulting impact on the mRNA.

A later study, in which not only the RNA levels were examined but also the corresponding protein levels, provided further insight into the role of asRNAs as regulatory switches. Their findings indicated that when asRNAs overlap the TSS, the introduction of a terminator, interrupting asRNA expression, significantly increases protein levels for 41 out of 152 genes, suggesting a primarily negative correlation between asRNA expression and protein output [60]. However, it is important to note that their studies did not provide definitive evidence of asRNA inhibition, as transcription may still initiate downstream of the introduced terminator. Nonetheless, in line with previous research, they concluded that asRNA transcription tends to silence low-expressed (noisy) genes without impacting highly expressed genes [10,34,60,65]. Interestingly, the genes that exhibit noisy expression levels were found to correlate strongly with asRNA expression. These asRNAs are generally transcribed over the TSS of the sense gene and are characterized by predominantly closed promoters, which suggests that they may be more susceptible to regulation by asRNA transcription. Notably, many of these genes are associated with stress responses, environmental adaptations, developmental processes or quiescence [27,65]. This observation indicates that regulatory genes are particularly prone to being modulated by this mechanism of asRNA transcription [27,34,65]. However, analyses of Net-seq and TT-seq data revealed that while asRNA transcription into the sense TSS is associated with histone modifications, this does not necessarily correlate with sense gene expression [28,40], suggesting a more complex interplay between asRNA transcription and gene regulation than previously understood. Closing of the promoter region does not necessarily represses transcription but could also mask repression sites resulting in the upregulation of the sense expression. The example of the *GAL10* lncRNA outlines an additional regulatory mechanism, as it can function in *trans* by forming R-Loop structures that result in faster induction of gene expression upon galactose induction [66,67]. This highlights how such structural formations could enhance the responsiveness of genes to environmental stimuli. However, these modes of action cannot be globalized and are even challenged by findings indicating that the *GAL10* lncRNA is rapidly released from the TSS rather than forming R-loops, as suggested by microscopy studies [63]. Possible effects of the asRNAs on the overlapping mRNAs are indicated in Figure 3.

This situation highlights the broader uncertainty about the global role of asRNAs, as genome-wide studies face challenges due to imprecise annotations and the variability in asRNA sizes across different conditions. Single-gene studies focusing on *PHO5*, *PHO84*, *GAL10* and *HMS2* revealed a complex picture [4,5,28,40,63,66,67]. In these studies, researchers frequently identify only small groups of genes fitting the demonstrated pattern, and the correlations between sense and asRNA levels often exhibit discordance, with some cases showing concordant levels. Moreover, for certain genes, there are even conflicting data regarding the relationship between sense and antisense transcripts such as for *GAL10*. This may indeed be the case, as the functional role of the asRNAs itself has often been insufficiently addressed in these studies. These post-transcriptional effects of asRNAs could provide an explanation for scenarios where the transcription rate remains unchanged while there are fluctuations in transcript levels. Such patterns suggest that asRNAs might influence events directly, such as processing, export or transcript stability rather than directly altering transcriptional activity [64]. Thus, all parameters need to be analysed before a conclusion is drawn to early. Nonetheless, it is crucial to note that a reduction in mRNA levels due to asRNA expression can correlate with an increase in the protein levels, as the mRNA has a reduced nuclear phase while being translated in the cytoplasm, reducing its overall half-life [64]. Therefore, all factors must be thoroughly analysed before reaching any premature conclusions.

## Cytoplasmic functions of lncRNAs and asRNAs in *S. cerevisiae*

The assumption that lncRNAs are mostly involved in nuclear functions has changed significantly due to findings that indicate the presence of XUTs and SUTs in polysome fractions [21,41,68]. This discovery suggests that these RNAs are actively engaged in translation processes. The ability of these lncRNAs to reach the cytoplasm might imply that they possess additional roles beyond their initial characterization for transcriptional leverages, since, if the RNA itself would be merely transient or non-functional, it should be subjected to degradation by the nuclear decay system. Supporting this notion, XUTs have been identified as targets of nonsense-mediated decay (NMD), a mechanism that typically recognizes and degrades faulty mRNAs present at the ribosome [41,42,68]. Moreover, studies in Mammalia further suggest that groups of antisense lncRNAs can influence translation initiation of the sense counterpart [6,11,69,70]. Thus, appearance of XUTs and SUTs at ribosomes might indicate additional roles, as otherwise their stabilization and export would be a waste of energy. While they may contribute to the post-transcriptional regulation of their complementary mRNAs, the recently proposed concept of pervasive translation offers an alternative explanation and importantly, both explanations do not exclude each other [20,21]. In fact, most XUTs are asRNAs and elegant experiments in which the RNAi system was reintroduced into *S. cerevisiae*, have demonstrated that ~80% of them form double-stranded RNA (dsRNA) structures with their regarding sense mRNA, which might be relevant during stress and cell cycle progression [41,71–74]. These findings indicate that asRNAs, particularly XUTs, are more likely to form double-stranded structures with their sense partners followed by SUTs, while only a few CUTs are double stranded.

Once the dsRNA reaches the ribosome, the strands are likely to be detached from each other, possibly by the ribosome. mRNAs are subsequently translated, while presence of small ORFs on asRNAs allows their pervasive translation which likely makes them susceptible targets for NMD. Additionally, XUTs with extended 3’UTRs were found to be Upf1 sensitive [41,42]. Upf1 is one of the main factors of NMD and the deletion of its gene results in an accumulation of NMD targets [75,76]. Consistently, lncRNAs have been identified as a major target of NMD in various organisms, with their sensitivity likely driven by the ‘faux 3’UTR’ model, whereby they are recognized due to inefficient translation termination caused by long 3’UTRs [77,78]. In addition, dsRNAs are enriched in mutants of ribosomal proteins or upon addition of the translational inhibitor cycloheximide [64]. The arrival of these dsRNAs at the ribosome explains why asRNAs are part of polysomes and can partly be translated when they comprise a smORF [21,41,68]. Whether the resulting peptides have functions or whether such asRNAs require just more time for NMD recognition is currently unclear.

## A general role for asRNAs in *S. cerevisiae* in boosting gene expression

Despite substantial research efforts, a general role for lncRNAs remained elusive. In particular because of their massive production, which possess the distinctive ability to generate dsRNA with their sense counterparts, they were expected to have a wider significance in cellular functions. Moreover, their decay through NMD already suggested that asRNAs may play a critical role in accompanying at least some of their corresponding mRNAs to the ribosome for translation. However, only in 2024 a series of experiments shed light onto a global function for asRNAs in *S. cerevisiae*. A cytoplasmic fractionation experiment that determined the compartmentation of the transcriptome highlighted an intriguing aspect of mRNA dynamics, revealing that a significant portion of bulk mRNAs is preferentially localized to the nucleus rather than the cytoplasm. Despite their primary function of being translated into proteins within the cytoplasm, the surprising finding that bulk mRNAs are predominantly nuclear suggested that the upstream processes, such as processing or export, are the rate limiting steps. Interestingly, it turned out that in contrast to that, asRNAs are in general more cytoplasmic and those mRNAs with a co-expressed asRNA were also more cytoplasmic while being present in a dsRNA [64]. This was shown in two ways: (a) with the data obtained in the RNAi experiments [41,64], but also for (b) an independent data set that captured dsRNAs, through RNA-co-immunoprecipitation (RIP) experiments with the J2 antibody [64]. This antibody detects RNA–RNA hybrids that are around 40 bp in length, thus sense/asRNA pairs [79,80]. Reassuringly, both methods highly correlated in the pool of ds forming RNAs (*r* = 0.72) and show a shift of the detected dsRNAs towards the cytoplasm as compared to ssRNAs [64].

**Figure 4.**
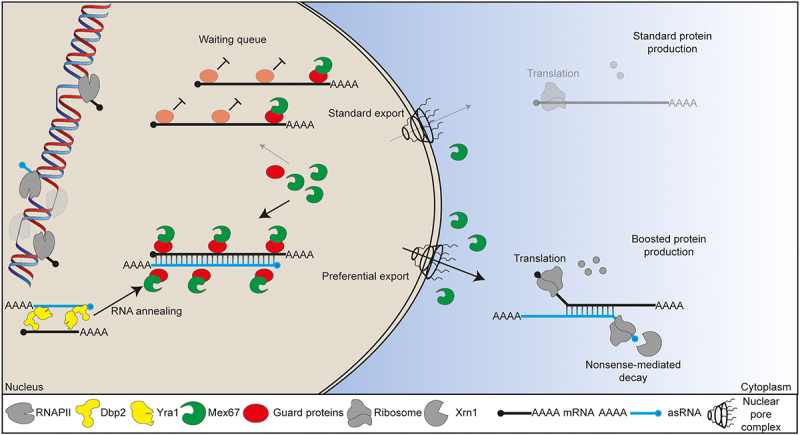
asRNA mediated boosting of gene expression. mRNAs are transcribed and wait after the guard protein-controlled processing in line for the recruitment of the export receptor Mex67-Mtr2 to leave the nucleus for translation. The transcription of asRNAs leads to the dsRNA formation with the sense counterpart facilitated by Dbp2 and Yra1. These dsRNAs bind Mex67 with a higher affinity and with more molecules leading to the preferential export into the cytoplasm where translation occurs. This process ultimately results in accelerated and increased protein production.

Generally, pre-mRNAs are retained in the nucleus, until they are properly processed. Maturation includes 5’ capping, splicing, 3’ cleavage and polyadenylation [81,82]. This is surveilled by the guard proteins, including Npl3, that monitors proper capping, Gbp2 and Hrb1, responsible for the control of splicing, Hrp1 that surveils correct 3’ cleavage and Nab2, which controls the attachment of the poly(A) tail [82–88]. These guard proteins share a common feature of operating through a switch-like mechanism. They recruit the export receptor heterodimer Mex67-Mtr2 (referred to as Mex67) to properly processed transcripts. However, they also retain defective transcripts in the nucleus until these are degraded by recruiting 5’and 3’ degrading enzymes instead of Mex67 [82]. Mex67 loading was furthermore shown to involve the TREX complex, composed of the THO-complex and Yra1 and Sub2 [89–91]. Interestingly, guard proteins are also present on sncRNAs and lncRNAs [54], which is not too surprising, as most of them are similarly processed like mRNAs. Thus, sense and asRNAs undergo similar maturation steps before they are exported. The transcriptome-wide compartmentation data suggested that dsRNAs are preferentially cytoplasmic compared to single stranded (ss)RNA. This phenomenon could be explained by an advantage of dsRNAs over ssRNAs during export (Figure 4). Indeed, further experiments revealed that dsRNAs are preferentially exported as shown by using the temperature-sensitive *mex67–5* mutant. At 37°C, RNA export is blocked leading to the accumulation of all transcripts in the nucleus [92]. After lowering the temperature to 25°C, Mex67 is re-activated, and transport begins. Strikingly, dsRNAs were shown to reach the ribosomes much earlier than ssRNA. Generally, export efficiency depends on the coverage of RNAs with Mex67 allowing passage through the nuclear pore complex [93,94]. A dsRNA would be expected to bind more Mex67 molecules than a ssRNA and *in vitro* studies showed that Mex67 has indeed a higher affinity towards dsRNAs and binds to it with twice the number of molecules [64]. This export assistance results in the faster translation of dsRNAs and thus to an accelerated protein production (Figure 4). Hence, the hidden power of asRNAs has the potential to push the nuclear export of their sense counterpart and in this way to boost their protein production. This suggests that asRNA possesses a general powerful function in yeast, which converts unimposing lncRNA into important regulators of gene expression. Future studies will characterize the sequence structure of dsRNAs and identify further associated proteins that might modulate these dynamics and will address whether such system is also relevant for other organisms.

RNA double strands are generated by the helicase Dbp2 in *S. cerevisiae* (Figure 4). Remarkably, cells deleted for *DBP2* are viable but show significant growth defects. Importantly, cells can no longer tolerate applied cellular challenges, such as changes in the salt concentration in the medium, resulting in cell death. This suggests that the expression of asRNAs and the subsequent formation of a dsRNA with the mRNA play a role in regulating expression programmes, with the asRNA acting like a VIP-ID. In fact, also in multicellular organisms certain asRNAs appear only upon developmental changes, which might suggest a general role for asRNAs in steering gene expression [7,8,12]. Clearly, asRNA and dsRNA increase upon changing environmental conditions, in particular different kinds of stresses [64]. Also, artificial expression of the bacterial RNAse III, which degrades dsRNAs, leads to a cell death when directed to the nucleus [64]. Thus, the asRNAs turned out to be the hidden leaders in gene expression for key situations. Extreme stress situations were shown to trigger a mechanism, by which the guard proteins and Mex67 dissociate from bulk mRNA allowing stress-specific mRNAs a preferred nuclear export upon direct Mex67 binding and without quality control by the guard proteins [87,88]. Whether these mostly heat shock protein chaperones encoding mRNAs form also dsRNAs with their asRNA remains to be shown.

Two helicases were shown to alter asRNA levels in cells: the DEAD-box helicase Dbp2 and the DExH-box helicase Mtr4 [64,95]. Such helicases were shown to be involved in all RNA-related processes such as transcription, RNA processing, RNP formation and translation [64,96,97]. Generally, they can promote RNA–RNA annealing or unwinding in an ATP-dependent manner. Dbp2 and Mtr4 were shown to be involved in lncRNA expression as loss of both helicases results in increased levels of XUTs and asRNAs [41,95]. However, Mtr4 is part of the TRAMP-complex, which recruits the nuclear exosome and thus is rather required for the nuclear degradation of asRNAs [64,98]. Nuclear accumulation of dsRNA and poly(A)^+^ RNA in *mtr4* mutants implies that dsRNA formation can occur, but that these RNAs await their degradation in the nucleus. Mtr4 is part of the TRAMP complex involved in RNA decay of ncRNAs but also of faulty mRNAs [87,88]. When such RNAs are part of a dsRNA, they might accumulate in *mtr4* mutants [64,99]. This accumulation of both, ss and dsRNAs, might lead to the titration of RNA processing and export factors, which could further interfere with RNA quality and restrict export. It is also conceivable that a surveillance mechanism for dsRNA formation exists that further fuels the accumulation of dsRNA in *mtr4* mutants. Thus, dsRNA formation is rather accomplished through Dbp2 activity as shown *in vivo* by J2 detection and *in vitro* with recombinant protein [64,100,101]. The protein is mainly localized to the nucleus, the compartment where dsRNA formation is required. However, some reports suggested also a possible cytoplasmic function for Dbp2 as it physically and genetically interacts with Upf1/UPF1, which might be important under certain conditions [95,102,103].

Yra1 can bind Dbp2 and inhibit its unwinding activity [100,101]. Thus, the presence of Yra1 might support the annealing activity of Dbp2. So far it was not shown whether dsRNA formation occurs co-transcriptionally or post-transcriptionally. Undirected post-transcriptional association of Dbp2 would cost important time and thus, hybridization should not be assumed to occur by chance. Interestingly, single gene studies using exogenous expression of an asRNA were able to direct preferential export, which indicates that the RNAs must not necessarily be expressed site by site to form a dsRNA. However, the plasmid encoded asRNA was highly expressed and thus present in high concentrations in cells [64]. Generally, Dbp2 can already bind co-transcriptionally to RNAs and is already present at the DNA [96,101,104]. Since also Yra1 binds co-transcriptionally to RNAs, it is likely that dsRNA formation can be already initiated co-transcriptionally; however, it remains to be determined how and when Dbp2 switches between its RNA unwinding and annealing activity. Yra1 (~25.4k) is present in cells in twice the amount of Dbp2 (~11.8k) [105] and it is conceivable that its presence rather determines which sense/asRNA pairs are annealed. Alternatively, it is possible that none of that is regulated, but rather the availability of the asRNA determines whether dsRNAs are formed.

Interestingly, Dbp2 was also shown to be involved in 3’ end formation of mRNAs and ncRNAs by providing a binding platform for Nrd1 [96]. This protein is known from NNS termination, which targets mostly NUTs but likely also CUTs directly after transcription for elimination through interaction with the TRAMP-complex and the nuclear exosome [37,38,44,51,52]. In this regard, it might also directly influence the formation of asRNAs [96]. As the loss of Dbp2 was additionally shown to influence R-Loop formation together with Sen1, the dsRNA forming enzyme could be a key player in modulating transcriptional induction of mRNAs and ncRNAs [66]. Dbp2 will certainly keep researchers busy as the regulatory mechanisms of dsRNA formation need further investigations.

## asRNAs in human: parallels to the asRNA-mediated gene boosting in yeast

asRNA-mediated gene boosting was shown to be a novel gene regulation layer in baker’s yeast [64]. Currently, it is unknown whether such a mechanism exists in other eukaryotes and multicellular organisms. While some arguments support its general existence, as outlined below, interference from other regulatory systems, like RNAi, complicates interpretations. Thus, future research is needed to clarify this.

The absence of the RNAi system in *S. cerevisiae* enabled the investigation of asRNAs in dsRNAs without the concern of them serving as degradation signals. Interestingly, closely related species such as *Schizosaccharomyces pombe* and *Naumovozyma castelli* are known to express similar lncRNA species, despite having a functional RNAi system [106–109]. Their transcriptomes include CUTs and XUTs, but they also have Dicer-sensitive transcripts known as DUTs. Notably, *N. castelli* only features a few DUTs and all correspond to XUTs, while more DUTs are found in *S. pombe* and those do not necessarily overlap with XUTs, indicating that some may be protected from Dicer by an as-yet-undefined mechanism [108,109]. One potential explanation might be the restricted subcellular localization of Dicer to specific nuclear regions in *S. pombe* [110]. Furthermore, in higher eukaryotes, asRNAs, commonly known as natural antisense transcripts (NATs), are expressed stably, suggesting that they can evade recognition by Dicer [12]. Thus, the presence of a functional RNAi system does not necessarily imply that asRNAs are unable to carry out similar post-transcriptional functions as discovered in *S. cerevisiae*. It is important to note that around 60% of all human genes are associated with annotated antisense transcripts, indicating that these transcripts might be hidden from the detection through the RNAi system. While lncRNAs and asRNAs show limited sequence conservation, similar functions have been observed across different organisms, including yeast, plants and mammals [12,15]. Consequently, it is likely that conservation is based on their genomic positioning and their ability to anneal with the regarding mRNA rather than on their individual sequences.

Traditionally, gene silencing was considered as one of the main function of NATs, as they can repress transcription through interference or target their sense counterparts for degradation via the RNAi system by forming endo-siRNAs, although such endo-siRNAs are relatively infrequent and restricted to certain cell types [111–113]. However, recent studies suggest that this might apply to a smaller subset of genes than previously thought, as NATs exhibit a broader spectrum of functions [8,10]. Depending on their genomic position regarding their sense gene, different roles have been attributed to NATs. Head-to-head oriented NATs are associated with transcriptional regulation, either repressing or promoting the expression of their sense gene, akin to the mechanisms observed in yeast. This regulation can occur through epigenetic modifications, transcriptional interference or also post-transcriptionally by affecting the protein expression through initiation of translation [8,11,69,70]. Interestingly, although it was reported earlier that NATs primarily exhibit a negative correlation with sense gene expression, more recent findings indicate that they may positively co-regulate these genes [15,114]. This outcome of more protein aligns with the general asRNA-mediated gene-boosting mechanism discovered in yeast.

In contrast to the head-to-head NATs, tail-to-tail overlapping NATs, or included NATs, are often associated with mRNA processing and stability (Figure 3). They regulate alternative polyadenylation, splicing and/or transcript stability, which have been implicated in cancer progression and other diseases, such as Alzheimer’s. This connection highlights their potential as therapeutic targets [8,12].

Interestingly, asRNA-mediated gene boosting has been shown to result in decrease of the corresponding mRNA levels in *S. cerevisiae* [64]. This may occur because the mRNA is degraded after a certain number of translation cycles, but its nuclear phase is decreased due to a preferential export. As a result, it seems that asRNAs induce the downregulation of their sense counterparts, which can be misleading as more protein is in fact the outcome of this asRNA-mediated boosting. Therefore, the observed effects of asRNA-mediated downregulation of sense RNA must be reconsidered, and one cannot automatically assume that decreased mRNA levels imply reduced protein levels. This phenomenon could potentially obscure important candidates in current studies.

In *S. cerevisiae,* asRNAs have a post-transcriptional role by guiding the mRNA to the ribosome, where translation occurs [41,64]. Since NATs feature complementary parts of the mRNAs in human cells, they can form dsRNAs with their respective counterparts. Thus, besides their roles in co-transcriptional regulation, they also impose the potential of the post-transcriptional regulation. To date, no direct role for NATs in mRNA export has been established. Beyond Dicer, a significant challenge for antisense–sense hybrids in human cells is that dsRNAs can activate an innate immune response; when present in the cytoplasm, they are detected by dsRNA sensors and subsequently targeted for elimination [12,115–118]. When the amounts are too high, apoptosis can be induced. However, sense and antisense RNA hybrids were found in the cytoplasm, suggesting that asRNAs might have adapted to these conditions and indicating that they likely serve roles and functions in this compartment [114]. It was suggested that endogenous dsRNAs can escape recognition by dsRNA sensors; however, it remains unclear how. One way might be RNA editing which allows escaping recognition of dsRNA by MDA5 [118–120]. Also, additional proteins, binding these dsRNA, might be involved to escape recognition. Finally, the gene boosting mechanism discovered in yeast was shown to be a quick response that resulted in the fast detachment of the dsRNA, possibly by this escaping from detection and degradation in human cells. Interestingly, J2 fCLIP-seq mainly identified mRNAs containing inverted Alu repeats (IRAlus) as a source of dsRNAs in human cells as they form strong secondary structures [121]. These IR-Alus are mainly present in introns and 3’ UTRs and are, among other functions, involved in post-transcriptional regulation as secondary structure formation is implicated in the nuclear retention of these RNAs [121,122]. Alternative polyadenylation can influence the inclusion or exclusion of these structures affecting the fate of the transcript [121]. The predominant presence of these structured elements complicates analysis and identification of sense/asRNA hybrids in human cells.

Importantly, although dsRNAs can trigger innate immune responses, some NATs were shown to affect expression of their complementary mRNA. NATs that overlap a small region of the 5’UTR and parts of the first exon of the mRNA and feature a 3’ inverted short interspersed nuclear element SINE B2 often in combination with an Alu element, have been shown to support ribosome association, known as SINEUPs. This enhancement occurs through their interaction with RNA-binding proteins and the presence of stem loop structures that serve as a ribosomal entry site [6,69,70,123]. SINEUPs range from 100 to 300 bases in length, and interestingly, while the expression of SINEUPs does not seem to alter the localization of the mRNA, they are found to be more abundant in the cytoplasm when paired with their sense RNA [70]. In contrast, NATs that contain embedded mammalian-wide interspersed repeat (MIR) motifs, referred to as MIR-NATs, have been shown to interfere with translation and play a role in proteostasis in neurodegeneration [11]. This illustrates that NATs can exert different effects depending on their associated secondary structures and/or bound proteins, which seem to be the major determinant of the regulative outcome. It remains unclear whether the formation of dsRNA occurs in the nucleus or in the cytoplasm for these translational regulatory mechanisms.

Given that the expression of lncRNAs is highly tissue-, developmental- and cell cycle-specific, it is probable that only few asRNAs are expressed under specific conditions in human cells compared to the relatively high number in yeast cells. Thus, careful selection of targets is necessary when analysing the potential effects on export. Notably, the expression of the antisense transcript TTN-AS1 associated with titin (TTN) has been shown to promote tumorigenesis by enhancing TTN expression [124]. This upregulation of TTN occurs not only at the transcriptional level but also by shifting the mRNA distribution more to the cytoplasm. Evidence for post-transcriptional effect of the asRNAs and their tissue or developmental specific appearance give rise to the assumption that asRNAs might be important to steer gene expression, not only in *S. cerevisiae* but possibly also in other eukaryotes. However, the functions of asRNAs in higher eukaryotes, as currently understood, do not exhibit a broad transferable character, impeded by the presence of the RNAi system, splicing of asRNAs and the fact that dsRNA triggers an innate immune response. Thus, some mechanisms must have changed during evolution. Nevertheless, some results suggest a transferable mechanism [11,123]. The tissue- and phase-specific expression patterns of lncRNAs, combined with the existence of different mRNA isoforms, complicate analyses. Indeed, experimental proof for such a general role as present in *S. cerevisiae* is currently missing in other species. Each sense/asRNA pair must be confirmed to form a dsRNA under specific conditions to establish a clear functional relationship. Furthermore, the presence of various dsRNA structures, such as IRAlus, adds another layer of complexity to these analyses. SINEUPs and MIR-NATs show that NATs can affect gene expression of the complementary mRNA and they seem to escape recognition by the innate immune response. Since their function depends on the overhanging sequence and the resulting bound proteins, it is possible that the significance of these sequences increased during evolution.

Despite these challenges and, at first glance, sometimes opposing findings, single-gene studies have demonstrated that the expression of NATs can lead to increased protein levels, as well as co-localization with their sense partners and accumulation of mRNAs in the cytoplasm, ultimately facilitating their recruitment to the ribosome. This offers a potential role for NATs in the export of mRNAs and the fine-tuning of their expression. Future research should focus on identifying sense-antisense pairs across different tissues and under different conditions, their localization and the resulting protein levels to learn more about the functional roles of asRNAs and their impact on gene expression and regulation, as well as mRNA and dsRNA dynamics within various biological contexts. To fully uncover the hidden power of asRNAs and to understand their roles and significance in cellular processes, further research is essential. Investigating the intricate mechanisms by which asRNAs influence gene expression will provide valuable insights into their contributions to both fundamental biology and disease states. As our understanding deepens, it may reveal novel therapeutic targets and innovative strategies for manipulating gene regulation. The exploration of asRNAs holds promise for advancing our knowledge of cellular dynamics and the underlying principles of life. Thus, the continued pursuit of this exciting area of research is critical for unlocking the full spectrum of functional diversity offered by asRNAs.
